# Review of Clinical Studies Targeting Inflammatory Pathways for Individuals With Autism

**DOI:** 10.3389/fpsyt.2019.00849

**Published:** 2019-11-25

**Authors:** Sina Hafizi, Dina Tabatabaei, Meng-Chuan Lai

**Affiliations:** ^1^Department of Psychiatry, Rady Faculty of Health Sciences, Max Rady College of Medicine, University of Manitoba, Winnipeg, MB, Canada; ^2^School of Medicine, Tehran University of Medical Sciences, Tehran, Iran; ^3^Centre for Addiction and Mental Health and The Hospital for Sick Children, Department of Psychiatry, University of Toronto, Toronto, ON, Canada; ^4^Autism Research Centre, Department of Psychiatry, University of Cambridge, Cambridge, United Kingdom; ^5^Department of Psychiatry, National Taiwan University Hospital and College of Medicine, Taipei, Taiwan

**Keywords:** autism, inflammation, neuroinflammation, autism spectrum disorder, immune system

## Abstract

Immune dysfunction and abnormal immune response may be associated with certain mechanisms underlying autism spectrum disorder (ASD). The early evidence for this link was based on the increased incidence of ASD in children with a history of maternal infection during pregnancy. Observational studies show increased prevalence of immune-related disorders—ranging from atopy, food allergy, viral infections, asthma, primary immunodeficiency, to autoimmune disorders—in individuals with ASD and their families. Evidence of neuroglial activation and focal brain inflammation in individuals with ASD implies that the central nervous system immunity may also be atypical in some individuals with ASD. Also, both peripheral and central inflammatory responses are suggested to be associated with ASD-related behavioral symptoms. Atypical immune responses may be evident in specific ASD subgroups, such as those with significant gastrointestinal symptoms. The present review aimed to evaluate current literature of potential interventions that target inflammatory pathways for individuals with ASD and to summarize whether these interventions were associated with improvement in autism symptoms and adaptation. We found that the current literature on the efficacy of anti-inflammatory interventions in ASD is still limited and large-scale randomized controlled trials are needed to provide robust evidence. We concluded that the role of immune-mediated mechanisms in the emergence of ASD or related challenges may be specific to subsets of individuals (e.g. those with concurrent immunological disorders, developmental regression, or high irritability). These subsets of individuals of ASD might be more likely to benefit from interventions that target immune-mediated mechanisms and with whom next-stage immune-mediated clinical trials could be conducted.

## Introduction

Autism spectrum disorder (ASD) is a neurodevelopmental condition characterized by early onset social-communication challenges, repetitive, stereotypical behaviors, and idiosyncratic sensory responsivity (1). Although several genetic and environmental factors have been linked to ASD, the underlying pathophysiology is still not well-understood (2). To date, although there have been great progress in developing behavioral and psychological support for individuals with ASD, no approved medication therapy exist for the core symptoms; established medication treatment aims at reducing concurrent challenges, such as increased irritability (3). Considering the heterogeneous nature of this condition and the paucity of biomarkers, development of a safe and effective “one-size-fits-all” medical therapy is challenging.

Several lines of evidence suggest a role of inflammation in the underlying developmental mechanisms of ASD (4–6). Epidemiological studies show an association between maternal infection [such as influenza (7) or cytomegalovirus (8)] during pregnancy and increased risk of offspring autism (9, 10). Also, family history of autoimmune diseases is associated with higher rate of ASD (11). In the animal models of ASD, offspring of animals with infection during pregnancy present physiological and behavioral alterations resemble ASD symptoms in human (12,13). People with ASD are suggested to have altered levels of inflammatory markers in which pro-inflammatory markers tend to increase while anti-inflammatory markers decrease (14, 15). Furthermore, postmortem studies of inflammation in the brain suggest alterations of brain immune response in ASD, including increased levels of proinflammatory markers (16) and increased microglial activation (17–19). Suzuki and colleagues showed increased microglial activation in the brain of individuals with ASD (20). Also, multiple studies on CSF and plasma/serum samples of individuals with ASD reported increased levels of proinflammatory markers (4, 21–23). Genetic studies reveal association between genomic variations in the immune-related genes and ASD (24). For example, human leucocyte antigen (HLA) DRB1 alleles are suggested to be associated with a higher risk of autism (25, 26). There is an emerging consensus that a better understanding of inflammation and its relation with the underlying pathophysiology of autism can provide new insight into the underlying mechanisms of the challenges faced by at least a subgroup of people with ASD (27), and help with developing new treatments. Given the current evidence, a growing body of literature has examined the role of medications with anti-inflammatory effects in the treatment of ASD-associated challenges, either alone or as an adjuvant therapy. In this narrative review, we aimed to summarize studies exploring the use of medications that target inflammatory pathways for people with ASD and evaluate the effectiveness and side effect profiles. Both medications with primary anti-inflammatory action (e.g. celecoxib) and those with additional anti-inflammatory properties beside their primary mechanisms of action (e.g. minocycline) have been included. These medications were selected based on the previous evidence indicating their anti-inflammatory properties in other psychiatric or medical disorders. Due to the availability of up-to-date meta-analysis on some of these agents, such as polyunsaturated fatty acids (28–31), they were not included in this review.

## Methods

This narrative review aimed to summarize the available evidence on the role of anti-inflammatory medications in the management of challenges associated with ASD. The literature search was performed using PubMed in July 2018. No publication date restriction was applied. We only included peer-reviewed studies on human subjects (with no age limitation) that were published in English language. The following search terms were used: (autism OR autistic OR autism spectrum disorder OR ASD OR pervasive OR pervasive developmental disorder OR pervasive developmental disorders OR Asperger OR Asperger’s) AND (celecoxib OR minocycline OR NAC OR N-acetylcysteine OR ACTH OR prednisone OR prednisolone OR hydrocortisone OR methylprednisolone OR dexamethasone OR cortisone OR triamcinolone OR betamethasone OR flavonoid OR lenalidomide OR pentoxifylline OR pioglitazone OR IVIG OR spironolactone OR topiramate OR memantine OR amantadine OR galantamine OR riluzole OR palmitoylethanolamide). We also identified additional articles by reference list/hand searching.

## Studies of Medications Targeting Inflammatory Pathways in People With ASD (In Alphabetical Order)

### Amantadine

Amantadine is an antiviral and anti-Parkinson medication that is widely used in the management of central nervous system disorders including multiple sclerosis (32) and traumatic brain injury (33). This medication is shown to be neuroprotective by the following mechanisms: (1) anti-inflammatory properties, mainly by inhibiting the release of proinflammatory factors (34, 35); (2) increasing the level of neurotrophic factors; and (3) inhibiting effect on N-methyl-D-aspartate (NMDA) receptors (34). In a placebo-controlled trial, King and colleagues studied the efficacy of amantadine in the management of ASD (36). They administered amantadine (n = 19) or placebo (n = 20) to individuals with ASD (age range of 5–19 years) over 4 weeks. The efficacy was measured using the Aberrant Behavior Checklist (ABC) and the Clinical Global Impression (CGI) scales. Amantadine group had slightly higher percentage of responders (equal or more than 25% reduction in irritability and/or hyperactivity according to parent-rated ABC). Based on the clinician-rated ABC, the amantadine group had significantly more improvement in absolute scores of hyperactivity and inappropriate speech. Also, amantadine group had higher improvement according to their CGI score. The side effects did not significantly differ between the amantadine and placebo groups.

Mohammadi and colleagues in a randomized controlled trial on children with severe behavioral issues (i.e. disruptive symptoms such as irritability) related to autism (age range of 4–12 years), compared risperidone plus amantadine (n = 20) with risperidone plus placebo (n = 19) over a 10-week period (37). They observed a significant improvement in hyperactivity and irritability as measured by ABC in the amantadine group as compared to placebo. Also, the amantadine group had significantly more improvement than the placebo group based on the CGI scores. Side effects were not significantly different between the groups.

### Celecoxib

Celecoxib is a nonsteroidal anti-inflammatory drug that selectively inhibits cyclooxygenase-2 (COX-2) enzyme. It has been widely studied as an adjuvant therapy in several psychiatric disorders including schizophrenia and depression (38, 39). In a randomized and double-blind controlled trial, Asadabadi and colleagues investigated the effect of celecoxib as an adjuvant therapy to risperidone in the treatment of children (age range of 4–12 years) with ASD and severe behavioral issues over 10 weeks. They used ABC rating scale to assess the clinical symptoms (40) (**Table 1**). Autistic children under treatment with celecoxib as an adjuvant therapy (n = 20) had significantly improved scores in irritability, social withdrawal/lethargy, and stereotypic behavior compared to the placebo group (n = 20). The study groups did not differ in terms of inappropriate speech, hyperactivity/non-compliance, or medications side effects.

**Table 1:** Summary of the studies on the role of anti-inflammatory medications in the management of ASD.

Drug	Participants	Type of study	Treatment	Outcomes	References	Benefit based on strength of evidence
**Amantadine**						
Amantadine	ASD (DSM-IV and ICD-10) N = 39 Age: 5–19 years	Double-Blind, Placebo-Controlled Trial Amantadine (n = 19), Placebo (n = 20)	Amantadine administered at 2.5 mg/kg/day for 1 week and then 5 mg/kg/day for 3 weeks.	Based on parent-rated ABC amantadine group had slightly higher (statistically non-significant) percentage of responders. However, based on the clinician-rated ABC, the amantadine group had significantly more improvement in absolute score of hyperactivity and inappropriate speech than placebo group. - Amantadine group had higher CGI score (not statistically significant) than placebo group.	King et al., 2001 (36)	Potential benefit
Amantadine plus risperidone	ASD (DSM-IV-R) and (6 or more DSM IV-TR symptoms) N = 39, Age: 4–12 years	Double-Blind, Placebo-Controlled Trial Risperidone plus Amantadine (n = 20) Risperidone plus placebo (n = 19)	Risperidone administered between 1 and 2.0 mg/day, Amantadine administered at 100 mg/d (if <30 kg) or 150 mg/d (if >30 kg), over 10 weeks	-Significant improvement in hyperactivity and irritability in the amantadine treated than the placebo group. - Significant improvement in the amantadine group on CGI	Mohammadi et al., 2013 (37)	
**Celecoxib**						
Celecoxib plus risperidone	Children with ASD ((DSM)-IV-TR)N = 40, Age: 4–12 years	Parallel-group, randomized, double-blind, placebo-controlled trial Risperidone plus Celecoxib (n = 20), Risperidone plus placebo (n = 20)	Celecoxib:100 mg/day to 200/300 mg/day Risperidone: 0.5 mg/day to 0.5 mg/week to 2–3 mg/day over 10 weeks	Significant improvement in: - Irritability -Lethargy/Social Withdrawal - Stereotypic Behavior as measured with ABC	Asadabadi et al., 2013 (40)	Potential benefit
**Corticosteroids**						
Chronic oral prednisolone treatment	A 6-year-old boy with autoimmune condition plus ASD	Case study	2 mg/kg/day for 10 weeks followed by 0.5 mg/kg every other day for 12 months 2 mg/kg of daily for 4 weeks, measured monthly by 0.5 mg/kg from weeks 4 through 12. Between weeks 12 to 28, alternate-day dosing was quantified in 0.25 mg/kg steps every 4 weeks.	Significantly improved: -Spontaneous speech, greater responsiveness to verbal communications (Token Test for Children), and improved social relatedness. -Receptive (Peabody Picture Vocabulary Test) and expressive Vocabulary -Visuomotor abilities and Performance IQ(WISC-R) -Decreased Stereotyped utterances (Diagnostic Checklist for Behavior-Disturbed Children)	Stefanatos et al., 1995 (46)	Unknown benefit
Low-dose steroid therapy	Autism with autoimmune lymphoproliferative syndrome (ALPS) Age: 18 months old	Case study	2 mg/kg/day for 10 weeks. the prednisolone dose was further reduced to 0.4 mg/kg every other day. Finally, the dose of 0.5 mg/kg every other day was the effective maintenance dose for treatment of the ASD and autoimmune condition	- Increased social interaction - Improvements in speech, gesturing, non-verbal communication, and language expression and comprehension subjective improvement, followed by objective improvement in speech and developmental milestones	Shenoy et al., 2000 (47)	
Steroid	Children with regressive Autism Spectrum Disorder (R-ASD) based on (DSM-IV) Steroid-treated R-ASD (STAR) (N = 20) Not-treated ASD patients (NSA) (N = 25), Age: 3–5 years	Retrospective Study	Oral prednisolone administered at 2 mg/kg/day. Treatment group: 4 Hz frequency modulated evoked response (FMAER) derived from language cortex of the superior temporal gyrus (STG)	-Significant increase in the 4 Hz FMAER spectral response and a significant reduction in response distortion compared to STAR group relative to the NSA group. - Significant improvement in STAR group subjects’ language ratings - Most STAR group children showed significant behavioral improvement after treatment. -STAR group language and behavior improvement were retained one year after treatment. - Groups did not differ in terms of minor EEG abnormalities. -Steroid treatment produced no lasting morbidity.	Duffy et al., 2014 (50)	
	Case 1: 4.5-year-old boy with Childhood disintegrative disorder (CDD) and generalized tonic clonic seizure Case 2: 4.5-year-old girl with CDD who was mildly encephalopathic	Case series	Case 1: Prednisolone (40 mg; 2 mg/kg/day for 2 weeks and then weaned by 5 mg a week over 8 weeks). Prednisolone (2 mg/kg for 2 weeks, tapered over 1 week	-Remained seizure free on Sodium Valproate (30 mg/kg/day) and at 2-year follow-up his behavior remained normal. -Normal academic school progress at 30 months follow-up. -Slow improvement with fewer periods of agitation in the next 3 weeks -Clear understanding of some verbal commands and developed a little speech. - No periods of agitation or ataxia,	Mordekar et al., 2009 (49)	
**Adrenocorticotropic hormone (ACTH)**						
ORG 2766	ASD (DSM-III) N = 14, Age: 5–13 years	Placebo-controlled double-blind cross-over trial	20 mg/day over 4 weeks period	Significant Improvement in irritability, stereotypic behaviors, hyperactivity, and excessive speech) as measured with ABC	Buitelaar et al., 1990 (45)	Potential benefit
ORG 2766	ASD N = 14 Age: 5–13 years	Double-blind, placebo-controlled cross-over trial	20 mg/day over 8 weeks period	- Significant Improvement in stereotypic behaviors - Social interaction-, play behavior, and stereotypy (P < 0.05 for each) compared with placebo (ABC and CGI); -Adverse effects were minimal	Buitelaar et al., 1992 (44)	
ORG 2766	ASD, N = 20 Age: 5–15 years	Controlled trial	40 mg/day for 8 weeks	-Significant improvement in the children’s play behavior and a significant increase in the social interaction between child and experimenter. -Gaze coordination between child and experimenter —Parents’ checklist ratings (ABC) as well as clinicians’ ratings (CGI).	Buitelaar et al., 1992 (43)	
ACTH	Case 1: 8-year-old boy with ASD Case 2: 2-year-old girl with ASD	Case studies	Case 1: prednisone l0 mg/day followed by ACTH 10 IU/day Case 2: 10 mg of prednisone plus ampicillin as prophylactic treatment daily for two months which was replaced by ACTH 10 IU i.m. daily	-He was attentive and had no more echolalia, or stereotypies, -He was able to communicate using simple phrases, and to perform simple tasks; to correctly use toys and was willing to play with other children -Improvement in pronouncing a few words, understanding demands, -Improved attentiveness, calmness, and less isolatedness.	Matarazzo et al., 2002 (48)	
**Flavonoids**						
	Children with ASD N = 37, Age: 4–14 years, 29 boys and 8 girls	Case studies	-luteolin (100 mg) + quercetin (70 mg) + Flavonoid (200 mg) −2 capsules/20 kg/weight, or at least 400 mg total flavonoid	-Significant improvement in bowel color, form and habits in 2–3 weeks (75%) -Significant reduction in Allergic-like symptoms in their skin -Significant improvement in eye contact, and attention to directions (50%) -Significant improvement in retained learned tasks and social interactions (30–50% of patients) -Significant improvement in speaking skills (10%)	Theoharides et al., 2012 (52)	Unknown benefit
	ASD children N = 40, Age: 4–10 years; 42 boys and 8 girls	Prospective, open-label trial Two age groups: 4–6 years (n = 25), 7–10 years (n = 25)	luteolin (100 mg/capsule, from chamomile) and quercetin (70 mg/capsule), and the quercetin glycoside rutin (30 mg/capsule) for 26 weeks	-Significant improvement in adaptive functioning as measured by Vineland Adaptive Behavior Scale (VABS) age-equivalent scores in the communication domain, daily living skills, and the social domain -Significant improvement in overall behavior as demonstrated by the decline in ABC subscale scores. -Age had no significant effect on results	Taliou et al., 2013 (53)	
	10-year-old boy	Case study	Co-Ultramicronized Palmitoylethanolamide/ Luteolin: 700 mg + 70 mg for 1 year	-Significantly decrease both total and subgroup scores, in particular; sociability, demonstrating improved behavioral outcome, in particular sociability (as per ATEC) -Significantly reduced most indexes of hyperactivity, as shown by reduction in motor stereotypies -Improved cognition as reported by parents and teachers (e.g. understanding of simple commands and accomplishing them easily; -Improved eye contact and the child’s behavior became more affectionate	Bertolino et al., 2017 (55)	
**Galantamine**						
	Autism (DSM-IV-TR) Case 1: 21-year-old male Case 2: a 32-year-old male Case 3: 42-year-old male	Case series	Case 1: Initial dose of 4 mg each month to a maximum of 12 mg as an adjunct treatment to his asthma medications Case 2: 4 mg daily Galantamine followed by a trial of donepezil due to side effects Case 3: Initial does of 4 mg per day for a month to a maximum of 16 mg	Case 1: Improved speech and cognition: active speech sound production -Spontaneous articulation, proper to the context and complex verbalizations Case 2: -Improvements in verbalizations, which were restricted to one- or two appropriate word responses to the questions asked Case 3: -Slight improvement in spontaneous speech and drooling -Significant improvement in aggressive behavior within the first month of treatment.	Hertzman et al., 2003 (57)	Potential benefit
	Children with autism N = 13, Mean Age: 8.8 +/- 3.5 years	12- week Open-label trial		-Significant improvement in irritability and social withdrawal subscales of ABC -Significant progresses in inattention and emotional liability (Conners’ Parent Rating Scale-R) -Improvement in the anger subscale of the Children’s Psychiatric Rating Scale.	Nicolson et al., 2006 (58)	
	ASD (DSM IV-TR) N = 40, Age: 4–12 years	Parallel-group, placebo-controlled, and double-blind trial Risperidone plus galantamine (n = 20) Risperidone plus placebo (n = 20)	Galantamine administered up to 24 mg/day or placebo, plus Risperidone administered up to 2 mg/day, for 10 weeks	-Significant improvement in the Irritability and Lethargy/Social Withdrawal subscales in the galantamine treated patients than the placebo group as measured with ABC	Ghaleiha et al., 2014 (59)	
**Intravenous immunoglobulin (IVIG)**						
	ASD (DSM-III-R) and immunological abnormalities N = 10, Age: 3–12 years	Open-label Study	400 mg/kg/month for 6 months at 4 weeks intervals	-Improvements in speech (better articulation and improved vocabulary) -one child almost completely recovered speech -Improved social behavior, better eye contact, loss of echolalia, and response to commands. - Mild improvement on spontaneous meaningful speech	Gupta et al., 1996 (60)	Potential benefit in individual with concurrent immunological disorder
	ASD (DSM-IIIR) N = 10, -Age: 4–17 years	Open-label study	4 infusions of (154 to 375 mg/kg), every 6 weeks	Mild improvements in attention and hyperactivity (n = 4) -No improvements (n = 5) - Almost total amelioration of autistic symptoms (n = 1)	Piloplys et al., 1998 (61)	
	ASD (DSM IV) N = 7 -Age: 3–6 years (6 male, 1 female),	Pilot Open Clinical Trial	400 mg/kg/month- for 6 months	Not beneficial for behaviors or severity	Delgiudice-Asch et al., 1999 (62)	
	Outpatient male children with autism (ICD-10), N = 12, Age: 4.2–14.9 years	Double-blind and placebo-controlled crossover study	0.4 g/kg at once	Significant improvement in Irritability, hyperactivity, Inadequate eye contact, and Inappropriate speech as measured with ABC	Niederhofer et al., 2003 (63)	
	ASD N = 26, Age: 3–17 years	Open retrospective study	400 mg/kg/month IVIG for 6 months	-Significant decrease in irritability, social withdrawal, stereotypy, hyper- activity, inappropriate speech as measured with ABC -Regressing to pre-IVIG level within 2 to 4 months of termination of IVIG (n = 22)	Boris et al., 2005 (64)	
	Children with ASD, N = 31	Open-label case series	Initiated with 2 g/kg monthly with 1 g/kg/day for 2 days monthly	Reports from parents: -Improvements in communication and/or language with fewer reporting -Improvements in aberrant behavior, repetitive behavior, and academics, social interactions, tics, motor function, and seizures -Significant Improvement in cognition and mannerisms on Social Responsiveness Scale, SRS) -Mild significant improvement in communication and motivation (SRS) -Significant improvement in irritability, lethargy/social withdrawal, hyperactivity, and inappropriate speech as measured with ABC	Connery et al., 2018 (65)	
**Lenalidomide**						
	ASD (DSM-IV-TR) N = 7, Age: 6–12 years	Open-label (Pilot Study)	2.5 mg/day for 12 weeks	-Significant improvement in socialization, expressive, and receptive language (CGI) -Significant decreased symptoms of autism based on the CARS scores in six children who completed the 6-week follow-up	Chez et al., 2007 (67)	Unknown benefit
**Memantine**						
	Children with ASD (DSM-IV-TR) N = 40, Age: 4–12 years	Double-blind, placebo-controlled study Risperidone plus Memantine (n = 20) Risperidone plus placebo (n = 20)	Risperidone was administered up to 3 mg/d and memantine was administered up to 20 mg/day Initiate Risperidone with dose of 0.5 mg with subsequent dose increase in 0.5 mg increments weekly Initiate Memantine with dose of 5 mg/day with subsequent dose increase in 5 mg increments weekly for 10 weeks	Significant improvement in irritability, stereotypic behavior, and hyperactivity as measured with ABC	Ghaleiha et al., 2013 (69)	Unknown benefit
	Autistic disorder, or Asperger disorder ((DSM-IV-TR) plus Moderate to severe ASD based on Social Responsiveness Scale-Adult Research Version (SRS-A), and the clinician-rated CGI N = 18, Age: 18–50 years	12-week, open-label treatment trial	Initiated with a daily dose of 5 mg that was increased by 5 mg weekly up to a maximum daily dose of 20 mg twice daily. (average dose, 19.7 ± 1.2 mg/day; range, 15–20 mg)	-Significant improvement in the severity of core features of autism based on (SRS) and (CGI) -Significant improvement in impaired reading and nonverbal communication based on Diagnostic Analysis of Nonverbal Accuracy Scale test and in executive function per self-report (Behavior Rating Inventory of Executive Functioning–Adult Self-Report Global Executive) -Significant improvement in cognitive dysfunction in particular executive areas of emotional control, task initiation, cognitive flexibility, self-regulation, planning and organization, response inhibition, and working memory; -Significant improvement in global functioning -Significant improvement in anxiety and ADHD symptoms	Joshi et al., 2016 (70)	
	ASD ((DSM-IV-TR), Autism Diagnostic Observation Schedule (ADOS) and the Autism Diagnostic Interview-Revised (ADI-R) N = 121, Age: 6–12 years Study 2: ASD (DSM-IV-TR) N = 66 (completed the study) Age: 5–16 years	Study 1: 12- week, randomized, double-blind, placebo-controlled, parallel-group. Placebo (n = 61), Memantine (n = 60). Study 2: 48-week open-label extension trial (enrolled participants n = 102; n = 66, completed the study) Placebo/Memantine (n = 35), Memantine/Memantine (n = 31)	Memantine doses were administered based on bodyweight and ranged between 3 and 15 mg/day	Study 1: -No significant between-group difference on the efficacy outcome of caregiver/parent ratings based on the Social Responsiveness Scale (SRS), -A Significant improvement as compared to baseline at Week 12 in both groups Study 2: -A tendency for improvement at the end of the extension period (48 weeks). -No significant improvements in the active group - Significant worsening of one communication measure in memantine group relative to placebo after 12 weeks.	Aman et al., 2017 (71)	
**Minocycline**						
	ASD (Autism Diagnostic Interview - Revised (ADI-R)), DSM-IV, or the Autism Diagnostic Observation Schedule N = 10 Age: 3–12 years	Open-label trial	1.4 mg/kg over 6 months	No clinical improvement noticed	Pardo et al., 2013 (73)	Unknown benefit
	ASD (DSM-IV-TR) N = 46	Randomized, double-blind placebo-controlled trial Risperidone plus minocycline (n = 23), Risperidone plus placebo (n = 23)	50 mg twice per day for 10 weeks plus Risperidone titrated up to 2 mg/day	-Significant improvement of irritability and hyperactivity/noncompliance	Ghaleiha et al., 2016 (74)	
**N-acetylcysteine**						
	Children with ASD (DSM-IV-TR), N = 29, Age: 3–12 years	Double-blind, randomized, placebo-controlled study NAC (n = 14) Placebo (n = 15)	Initiated with 900 mg/daily for 4 weeks, followed by 900 mg/twice daily for 4 weeks and then 900 mg/three times daily for 4 weeks	-Significant reduction in irritability in the treatment group as measured with ABC -Significant improvement on stereotypies as measured with RBS-R - Significant improvements in social cognition and autism mannerisms as measured with SRS	Hardan et al., 2012 (80)	Potential benefit
	8-year-old boy with ASD (DSM-IV)	Case study	800 mg per day over 6 weeks	- Significant reduction in his nail-biting behavior - Significant reduction in his autistic symptoms 1 month after the onset of treatment -Significant improvement in: social interaction ( visual analog scale), verbal skills and communication (visual analog scale), aggressive behavior, hyperactivity and limited interests, and severity and frequency of his blinking tic (parents report)	Ghanizadeh et al., 2012 (78)	
	Children and adolescents with ASD (DSM-IV-TR), N = 40, Age: 3.5–16 years	Randomized double blind placebo controlled trial NAC plus Risperidone (n = 17) Placebo plus Risperidone (n = 14)	1200 mg/day NAC + Risperidone or placebo + Risperidone for 8 weeks	Significant improvement in irritability as measured with ABC	Ghanizadeh et al., 2013 (81)	
	4-year-old boy with self-injurious behavior	Case study	Initiated with 0.45 g/d and titrated up to 1.8 g/day over 3 weeks	Improvement in frequency and severity of self-injurious behavior	Marler et al., 2014 (77)	
	17 years old with ASD	Case study	Administered 20% acetylcysteine oral solution started at 600 mg twice daily as an add-on to Quetiapine therapy for six weeks. continued acetylcysteine with 900 mg twice a day plus Quetiapine 200 mg twice a day	Significantly improved tantrums, irritability, and aggressive behavior	Stutzman et al., 2015 (79)	
	Children with autism spectrum disorder (ASD) N = 40, Age: 4–12 years	A Randomized, Double-Blind, Placebo-Controlled Clinical Trial Risperidone plus NAC (n = 20) Risperidone plus placebo (n = 20)	Risperidone administered up to 1 and 2.0 mg/d, NAC dosage was 600 to 900 mg/day over 10 weeks	-Significant interaction of time and treatment on irritability, and hyperactivity/ Noncompliance subscales. -By the end of trial, the treatment group had more improvement in irritability and hyperactivity/ noncompliance subscales scores as measured with ABC	Nikoo et al., 2015 (82)	
	Children with Asperger’ s disorder, PDD NOS, ASD ((DSM-IV, (ADI-R))) N = 31, Age: 4–12 years	Randomized, double-blind, placebo-controlled trial NAC (n = 16), Placebo (n = 15)	Regular daily dose was 56.2 mg/kg over week 12, with dose varying between 33.6 - 64.3 mg/kg. Initiated with the dose of 300 mg/day for weights between 15 to 30 kg Initiated with the dose of 600 mg/day, > 30 kg	- No significant difference between the treatment and placebo groups on the CGI test	Wink et al., 2016 (83)	
	Children with ASD (DSM-IV-TR) N = 98, Age: 3–9 years	Placebo-controlled, randomized clinical trial NAC (n = 48) Placebo (n = 50)	500 mg/day orally administered over 6 months	-No significant differences between treatment and placebo-treated groups for, scores on the SRS, Children’s Communication Checklist and the RBS -No significant difference found on the three global impression scales: DBC-P, CGI, and PGI-I	Dean et al., 2017 (84)	
**Palmitoylethanolamide**						
	Case 1: 13-year-old male with autism Case 2: 15-year-old male with autism	Case series	Case 1: Oral administration of 1/2 tablet (300 mg) twice daily, followed by (600 mg) tablet twice daily for a month Case 2: 600 mg tablet for 3 months	Case 1: -Improvement in his behavior and expressive language (parents and school teacher’s reports) -Decrease in tantrums, outburst, self-talking, and stereotypies. -Improvement in skin eczema, nose-picking, asthmatic cough, and allergy stigmata Case 2: -Improvement in speech, sociability, sensory/cognitive, and overall behaviour -Improvement in aggression and cognitive and behavioral skills, and Language	Antonucci et al., 2015 (87)	Potential benefit
	Autistic children (DSM-V) N = 62, Age: 4–12 years	Randomized, parallel group, double-blind placebo-controlled trial Risperidone plus PEA (n = 31), Risperidone plus placebo group (n = 31)	Children in both groups received Risperidone equally with initial dose of 0.5 mg and stepwise 0.5-mg weekly increases for the first 3 weeks plus 600 mg PEA twice daily over 10 weeks	-Significant improvement on ABC irritability and hyperactivity/noncompliance, and inappropriate speech symptoms related to risperidone plus placebo group	Khalaj et al., 2018 (88)	
**Pentoxifylline**						
	Behavioral anomalies of biological basis or autism N = 36, Age: 3–15 years	Open-label study	150–600 mg/day over 1 month	-Remarkably effective in (N = 10) Fairly effective in (N = 8) Slightly effective in (N = 3) No effect (N = 2) -Improvement in sameness maintenance syndrome as well as in increasing the understanding of language and human relations and in their self-image to the extent (based on authors observations)	Sogame et al., 1978 (90)	Potential benefit
	ASD N = 30 Case reports: aged 12, 13, and 15 years	Open-label study	Not specified	-Marked improvement (n = 6) -Slight amelioration of symptoms (n = 14) -three out of six patients reported marked improvement	Nakane et al., 1980 (91)	
	Male autistics N = 20, Age: 3–22 years	Open-label study	200 mg/day for 3 months	–35% improvement in behavior and mental development	Shimoide et al., 1981 (92)	
	Psychotic (N = 18) and autistic children (N = 2): a 5-year-old boy and a 7-year-old girl	Open-label study	Dose of 50 mg/day to 200 mg/day Over 4–10 months	-Significant improvement in pronunciation of syllables and words -Significant improvements in behavior and in language -Significant Increased in attention to others and speech -Improvement in pronunciation of syllables and words	Turek et al., 1981 (93)	
	ASD (based on DSM IV-TR) N = 40, Age: 4–12 years	Randomized double-blind, placebo-controlled Risperidone plus pentoxifylline (n = 20), risperidone plus placebo (n = 20)	Pentoxifylline: 200- 600 mg/day Risperidone: 0.5- 3 mg/day Over 10 weeks	Significant improvement in: -Irritability, social withdrawal, and stereotypic behavior -Hyperactivity and inappropriate speech As measured with ABC	Akhondzadeh et al., 2010 (94)	
**Pioglitazone**						
	ASD (DSM-IV) N = 25, Age: 3 –17 years	Open-label study	30 mg/day for individuals with age range of 3 to 5 years 60 mg/day for individuals with age range of 6–17 years -Over 4 months	-Significant decrease in hyperactivity, Irritability, lethargy, and stereotypy as measured with ABC	Boris et al., 2007 (98)	Potential benefit
	Outpatients with ASD N = 40, Age: 4–12 years	Randomized, double-blind, parallel-group, placebo-controlled trial Risperidone plus Pioglitazone (n = 20) Risperidone plus placebo (n = 20)	Risperidone: initial dose of 0.5 mg/day which was titrated in 0.5 mg increments every week over 10 weeks Pioglitazone: dose of 30 mg/day (15 mg two times daily) in one group	-Significant reduction in irritability, lethargy/social withdrawal and hyperactivity/non-compliance	Ghaleiha et al., 2015 (99)	
**Riluzole**						
	ASD (DSM-IV-TR), N = 40, Age: 5–12 years	Double-Blind, Placebo-Controlled, Randomized Trial Risperidone plus Riluzole (n = 20), Risperidone plus placebo (n = 20)	Riluzole administered up to 50 or 100 mg/day according to bodyweight. Risperidone administered up to 2 or 3 mg/day (according to bodyweight) for 10 weeks.	-Significant improvement in the irritability, lethargy/social withdrawal, stereotypic behavior, and hyperactivity/non-compliance subscale in Riluzole-treated patients - 11 individuals in Riluzole group and 5 individuals in the placebo group were categorized as responders according to their CGI scores	Ghaleiha et al., 2013 (110)	Potential benefit
**Spironolactone**						
	12-year-old boy with well-established autism, immune dysregulation, and food allergies	Case Report	2 mg/kg/day for 4 weeks	-Significant improvement in irritability, social withdrawal, stereotypy, hyperactivity, inappropriate speech as measured with ABC -Significant improvement in receptive language (as measured with Peabody Picture Vocabulary Test III)	Bradstreet et al., 2007 (103)	Unknown benefit
**Topiramate**						
	Autistic children (DSM IV), N = 40, Age: 3–12 years	Double-blind, placebo-controlled trial Risperidone plus Topiramate (n = 20) Risperidone plus placebo (n = 20)	Risperidone was administered up to 2 mg/d for children between 10 and 40 kg and 3 mg/day for >40 kg. Topiramate was administered up to 100 mg/day for individuals <30 kg and 200 mg/day for individuals >30 kg) over 8 weeks	Significant improvement on irritability, stereotypic behavior, and hyperactivity/non-compliance subscales as measured with ABC	Rezaei et al., 2010 (107)	Potential benefit

### Corticosteroids and ACTH

Corticosteroids have been effectively used in the management of neurological disorders, such as Landau–Kleffner syndrome (LKS) (41). Due to similarities in the progression and development between LKS and *regressive* ASD, and considering the growing evidence on the link between inflammation and ASD, a number of studies have investigated the effects of corticosteroids or adrenocorticotrophic hormone (ACTH) in regressive ASD (42). In a series of trials, Buitelaar and colleagues studied the role of ORG 2766, an ACTH analog, in the management of children with ASD (43–45). In the first trial, they enrolled 14 children (age range of 5–13 years) with ASD in a double-blind crossover study (45). ORG 2766 was administered over a 4-week period. They reported significant improvement in clinical symptoms (i.e. irritability, stereotypic behaviors, hyperactivity, and excessive speech) as measured by the parent-reported ABC and playroom data. In the second crossover trial, they reported positive effects of ORG 2766 (administered over an 8-week period) on symptoms of 20 children (age range of 5–15 years) with ASD as measured by ABC and CGI (43). In their third study, they reported the effect of ORG 2766 on social interactions of autistic children enrolled in their first trial (44). They found that ORG 2766 therapy resulted in a significant increase in the quality and quantity of social interactions in the participants.

In a single-case study, Stefanatos and colleagues administered corticosteroid (i.e. prednisone) to a 6-year-old boy with regressive ASD over a 28-month period (46). The patient started losing his language abilities at age 22 months. The medical and neurological assessments were mostly unremarkable except for hypoperfusion of perisylvian cortical region in SPECT and abnormal steady-state auditory evoked potentials. Corticosteroid therapy resulted in significant improvement in language, social abilities, and stereotypic behaviors.

More recently, Shenoy and colleagues reported a case of regressive ASD that was diagnosed at the age of 18 months (47). He presented with progressive lymphadenopathy, microcytic anemia, mild thrombocytopenia, and low white blood cells count. He was started on corticosteroid at the age of 33 months. After about a month of steroid therapy, the patient started regaining his language and communication abilities. After 26 months of therapy, all the laboratory values returned back to normal.

Matarazzo described two cases of regressive ASD with histories of recurrent bacterial infections (48). Both individuals were initially started on corticosteroid therapy and later due to the side effects, were switched to ACTH. In both cases, corticosteroid therapy led to improvement in language and communication skills, as well as behavioral symptoms, such as stereotypic behaviors.

Mordekar and colleagues reported two cases of regressive ASD treated with corticosteroid (49). The first case was a 4.5-year-old boy with ASD and generalized tonic–clonic seizure that regained his personality and language abilities after treatment with corticosteroid. The second case was a 4-year-old girl with ASD who lost her language and communication abilities after experiencing neurological symptoms associated with ataxia and fluctuation of consciousness. Her symptoms started to improve after three weeks of treatment with prednisolone. Forty-eight months after treatment, she was back to her normal function and personality.

Most recently, a retrospective study (age range of participants, 3–5 years) compared 20 children with regressive ASD treated with corticosteroids (for a maximum period of 4 months) with 24 autistic children without corticosteroid treatment; the authors observed significant improvement in the frequency modulated auditory evoked response, language function, and ASD symptom score according to DSM-IV criteria after corticosteroid therapy (50).

Taken together, there is a lack of randomized controlled trials to inform the clinical utility of corticosteroid in individuals with ASD. Single case, case series, and observational studies indicate the potential of corticosteroid or ACTH as a treatment for *regressive* ASD, but this awaits rigorous controlled trials to validate and to investigate the potential underlying mechanisms of action.

### Flavonoids

Flavonoids are a large group of nutrients found in vegetables and fruits. They are shown to have anti-inflammatory effects through inhibition of inflammatory pathways, including the synthesis/activity of c-reactive protein, proinflammatory cytokines, and microglia (51). Theoharides and colleagues studied the effects of a flavonoids mixture (luteolin, quercetin, and rutin) in an open-label case-series of 37 children (age range of 4–14 years) with ASD over a period of 4 months (52). They reported improvement in attention and eye contact (50%), allergy and gastrointestinal symptoms (75%), and social interaction (25%). No side effects due to the treatment were noted. More recently, using similar supplement, Taliou and colleagues in an open-label trial studied 40 children (age range of 4–10 years) with ASD over a 26-week period (53). They reported significant improvement in communication, daily living skills, and social skills as measured by the Vineland Adaptive Behavior Scales (VABS) and also in hyperactivity, irritability, lethargy, and stereotypical behavior as measured by the ABC. More recently, the same group using the same database reported that children with ASD that had the most behavioral improvement were the ones that had reduction in the serum levels of IL-6 and TNF following treatment with luteolin (54). Most recently, Bertolino and colleagues reported a single case of a 10-year-old boy with regressive ASD and history of recurrent febrile seizure who underwent treatment with a flavonoid supplement (Palmitoylethanolamide/Luteolin) over a 12-month period (55). Treatment with the flavonoid supplement led to improvement in ASD symptoms as measured by ASD treatment evaluation checklist (ATEC) and a scale for stereotypic behavior and the frequency of enuresis. No side effects were reported.

Overall, there may be a potential for the use of flavonoid in the management of individuals with ASD that need to be further examined in robust clinical trials. Due to insufficient evidence, no clinical recommendations can be made so far with regard to the use of flavonoids.

### Galantamine

Galantamine is an acetylcholinesterase inhibitor approved for the treatment of Alzheimer’s disease. It also has anti-inflammatory properties by inhibiting the pro-inflammatory markers, such as TNF and cytokines (56). Hertzman studied the effect of galantamine on three adults with autism (57). The first patient was a 21-year-old male. Treatment with 4 mg of galantamine for a month led to improvement in verbal communication and the patient started making sound and responding. The second case was a 32-year-old male that after treatment with 4 mg of galantamine presented with improvement in verbalization; however, he experienced allergic reactions and the medication was discontinued. The third case was a 42-year-old male. He presented aggressive behaviors and never had verbal communication. Treatment with 4 mg of galantamine within a month led to reduction of aggressive behaviors. In a more recent study, Nicolson and colleagues studied galantamine effects in an open-label trial on 13 children (mean age, 8.8 ± 3.5 years) with autism over a 12-week period (58). Children presented significant improvement in irritability and social withdrawal as measured by ABC and emotional liability and attention as measured by Conners’ parent-rating scale. Moreover, children presented improvement in aggression as measured by the children’s psychiatric rating scale. Except for headache in one child, galantamine treatment was well tolerated. In the only randomized controlled trial available, Ghaleiha and colleagues compared risperidone plus galantamine (n = 20) with risperidone plus placebo (n = 20) over a 10-week period in children (age range of 4–12 years) with ASD (59). They observed a significant improvement in irritability and lethargy/social withdrawal subscales of ABC with galantamine as compared to placebo. The side effects were not significantly different between the groups. These initial findings indicate the potential to further evaluate the benefit and safety of galantamine for autistic individuals in robust clinical trials.

### Intravenous Immunoglobulin

Several open-label trials have investigated intravenous immunoglobulin (IVIG) therapy in ASD, particularly those with concomitant immunological deficits. Gupta and colleagues administered IVIG to 10 children (age range of 3–6 years) with ASD and IgG deficiency and/or high levels of maternal rubella antibody at 4 weeks interval for a minimum period of 6 months (60). Behavior, cognition, and developmental characteristics of these children were evaluated using Peabody picture vocabulary test, the VBAS, skill evaluation, and preschool language test. Treatment with IVIG resulted in improvement in social behavior, eye contact, echolalia, response to commands, and speech.

Later, Plioplys and colleagues studied IVIG administration in 10 children (age range of 4–17 years) with ASD and immunologic abnormalities (61). The IVIG infusions were administered every 6 weeks. Six children received four infusions and one individual each received 1, 3, 5, and 6 infusions. While five individuals did not experience any changes in their symptoms, four children were reported by their parents to have mild improvement in attention and hyperactivity. One individual had a significant improvement in social behavior and language in a step-wise fashion after each infusion. However, the clinical improvement reversed 2 months after the last (6th) infusion, and he again presented with the same severe autistic symptoms as he presented before participating in the study.

DelGiudice-Asch and colleagues administered IVIG to seven children (age range of 3.5–6 years) with ASD without known immunological anomalies at monthly intervals over a 6-month period (62). Five children received the six infusions and finished the study. Overall there were no significant improvement in any of the clinical symptoms as measured by Ritvo-Freeman Real Life rating scale, the children Yale-Brown Obsessive-Compulsive Scale, CGI scale for autistic disorder, and the ASD modification of the NIMH global obsessive-compulsive scale.

In a double-blind, placebo-controlled crossover study, Niederhofer and colleagues studied the effects of a single dose of IVIG on clinical symptoms of 12 children (age range of 4.2–14.9 years) with ASD as measured by ABC (63). The participants were medication free and *had no immunological disorders*. They found a significant improvement in parent-reported irritability, hyperactivity, inadequate eye contact, and inappropriate speech as measured by ABC and drowsiness and decreased activity as measured by the symptom checklist. However, none of the clinician ratings showed significant differences between placebo and IVIG groups. No remarkable side effects were noted.

In an open-label study, Boris and colleagues administered IVIG to 26 children (age range of 3–17 years) with ASD (with history of developmental regression) at a monthly basis for 6 months (64). They observed a significant improvement in total aberrant score (37%), hyperactivity (39%), inappropriate speech (25%), irritability (42%), lethargy (35%), and stereotypic behavior (28%) on the ABC. Only a small number of the participants experienced side effects. However, most children’s behavior returned to their pre‐IVIG status within 2–4 months of discontinuing the IVIG.

In an open-label case-series study, Connery and colleagues administered IVIG to 31 children (mean age of 9 years and 9 months with standard deviation of 4 years and 5 months) with ASD and autoimmune encephalopathy on a monthly basis (65). Clinical outcomes were measured using ABC and the Social Responsiveness Scale (SRS) as completed by the caretakers. IVIG treatment resulted in significant improvement in cognition and mannerism sub-scales and the total score of SRS. Moreover, they reported a significant improvement in irritability, lethargy, hyperactivity, and speech sub-scores as well as the total score of ABC. The most common side effects were headache and vomiting, which were common but limited to the infusion period.

Taken together, current evidence suggests potential benefit of IVIG in children with ASD and concomitant immunological disorders, such as autoimmune encephalopathy; however, given the common side effects and the invasiveness of the intervention, IVIG treatment needs to be considered cautiously, weighing the risks and benefits. More robust clinical trials in large cohort are required to further establish the evidence. For children with ASD *without* known immunological deficits, current evidence is inconsistent and insufficient, and therefore, there is no clear rationale so far to recommend IVIG treatment for this subpopulation of ASD.

### Lenalidomide

Lenalidomide, a derivative of thalidomide, is an immunomodulatory medication widely used in the treatment of hematologic disorders and malignancies (66). In an open-label study, Chez and colleagues administered lenalidomide to seven male children aged 6 to 12 years with ASD and history of developmental regression and elevated levels of TNF-alpha, for a 12-week period (67). They observed a significant improvement in ASD symptoms as measured by the Childhood Autism Rating Scale (CARS) and also in expressive language as measured by CGI. Moreover, although not significant, treatment with lenalidomide decreased the levels of TNF-alpha in both serum and CSF. However, it is important to note that among the seven children enrolled, two developed a rash and discontinued the study, and an additional one discontinued from the study after eight weeks due to transient drop of absolute neutrophil count to <1500.

### Memantine

Memantine is an NMDA receptor blocker with suggested neuroprotective and anti-inflammatory effects (68). Ghaleiha and colleagues in a randomized controlled trial compared treatment with memantine plus risperidone (n = 20) with risperidone plus placebo (n = 20) in children (age range of 4–12 years) with ASD (69). They reported significant improvements in irritability, stereotypic behavior, and hyperactivity/non-compliance as measured by ABC in the memantine group compared to the placebo group. They did not find any significant difference in the side effects between groups. More recently, an open-label study evaluated the efficacy and tolerability of memantine on 18 adults (age range of 18–50 years) with autism over a 12-week period (70). Treatment with memantine was associated with significant improvement in autism severity as measured by the SRS, CGI, MGH ASD rating scale, and brief psychiatric rating scale as well as anxiety, ADHD symptoms, nonverbal communication (measured by Diagnostic Analysis of Nonverbal Accuracy Scale test) and in executive function (measured by the Behavioral Rating Inventory of Executive Functioning and Cambridge neuropsychological test automated battery). Treatment with memantine was not associated with any serious adverse event. Most recently, in the largest randomized controlled trials so far on the efficacy of memantine in children (age range of 6–12 years) with autism, Aman and colleagues compared treatment with memantine (n = 60) with placebo (n = 61) over a 12-week period (71). They did not observe any significant clinical improvement as measured by the SRS, although in the 48-week open-label follow up, a trend of improvement was observed. They reported two serious adverse events during the study that both were recognized to be unrelated to the treatment.

Taken together, the current evidence does not support a role for memantine in the treatment of core autism characteristics and associated challenges. It is notable that some large-scale randomized controlled trials are still ongoing (e.g. https://clinicaltrials.gov/ct2/show/NCT01972074).

### Minocycline

Minocycline is a tetracycline-class antibiotic with anti-inflammatory effects (72). In an open-label trial, Pardo and colleagues investigated the effect of minocycline on clinical measures (as measured by CGI and VABS) and blood/cerebrospinal fluid (CSF) growth factors and markers of inflammation in autistic children (age range of 3–12 years) with history of regression (n = 10) over a 6-month period (73). While minocycline did not have a significant effect on core autism symptoms and adaptive functioning, it significantly reduced IL-8, an anti-inflammatory cytokine, and brain-derived neurotrophic factor (BDNF) in the CSF, and BDNF level (as normalized by alpha-2 macroglobulin level) in serum. In contrast to BDNF, hepatic growth factor (HGF) in the CSF significantly increased after treatment with minocycline.

In a randomized placebo-controlled trial, Ghaleiha and colleagues evaluated the effect of minocycline as an adjunctive therapy to risperidone in autistic children over a 10-week period (74). Minocycline significantly improved scores on irritability and hyperactivity/noncompliance on the ABC; however, it did not have any significant effect on lethargy/social withdrawal, stereotypic behavior, and inappropriate speech. The authors did not report any significant difference in side effects between groups. The observed difference in the effects of minocycline on clinical presentations of the participants in the above studies might be due to the difference in the administered dosage, 1.4 mg/kg/day in the Pardo et al. study (73) versus 100 mg/day in the Ghaleiha et al. study (74).

Taken together, there is still insufficient evidence to support the use of minocycline in the treatment of core autism symptoms or associated challenges.

### *N*-Acetylcysteine

There is a growing interest in the potential use of N-acetylcysteine (NAC) in the treatment of psychiatric disorders (75). NAC acts as a precursor for glutathione, the most abundant antioxidant in the brain and is shown to have both anti-oxidant and anti-inflammatory properties in the body (76). Several case reports and trials have investigated the effect of NAC in individuals with ASD.

In a case study, Marler and colleagues administered NAC to a 4-year-old autistic child with self-injurious behavior over a 3-week period and observed improvement in frequency and severity of self-injurious behavior (77). In another case study, Ghanizadeh and Derakhshan examined NAC effects on clinical symptoms of an 8-year-old boy with ASD over a 6-week period (78). They observed significant improvement in social impairment, social interaction, nail-biting behavior, verbal skills and communication, tics, and aggression as measured with visual analog scale by the parents. Stutzman and Dophiede studied a 17-year-old boy with ASD and intellectual disability who was treated with NAC as an adjuvant therapy to quetiapine over 6 weeks (79). Treatment with NAC led to a reduction in irritability and aggressive behaviors.

Several small-scale clinical trials suggest a potentially positive role for NAC. Hardan and colleagues in a 12-week randomized controlled trial treated children (age range of 3.2–10.7 years) with ASD with NAC (n = 14) or placebo (n = 15) (80). They observed a significant improvement in irritability (as measured by the ABC) and a trend toward significance on stereotypic/repetitive behaviors (as measured by ABC and Repetitive Behavior Scale-Revised) in the NAC compared to the placebo group. Additionally, NAC significantly improved mannerism score (as measured by the SRS). Groups did not significantly differ in terms of medication side effects. In a more recent randomized controlled trial, Ghanizadeh and colleague observed a significant improvement in irritability score (as measured by ABC), but not the core autism symptoms, in children (age range of 3.5–16 years) with ASD treated with risperidone and NAC (n = 17) as compared to risperidone and placebo (n = 14) over an 8-week period (81). In this trial, the most commonly reported side effects were constipation, increased appetite, fatigue, nervousness, and daytime drowsiness. In a similar study, Nikoo and colleagues compared autistic children (age range of 4–12 years) under treatment with risperidone and NAC (n = 20) with those treated with risperidone and placebo (n = 20) over a 10-week period (82). They observed a significant improvement in irritability and hyperactivity/noncompliance as measured by the ABC. No significant differences in side effects were found between the groups. Wink and colleagues in a 12-week randomized controlled trial compared autistic children (age range of 4–12 years) on NAC (n = 16) versus placebo (n = 15) (6 patients were later excluded due to adverse effects or losing follow up) in terms of their social impairment as measured by CGI scale, and also oxidative stress markers in the blood (83). They did not find any significant difference between groups with regard to their CGI, ABC, SRS, or VABS-II scores. However, they observed a significant increase in the level of glutathione and a trend toward significance for increase in the level of oxidized glutathione (GSSG) in the NAC group.

Dean and colleagues in a randomized controlled trial compared treatment with NAC versus placebo in 98 children (age range of 3.1–9.9 years) with ASD (50 in the placebo group, 48 in the NAC group) (84) and observed no significant effect of NAC on their primary (i.e. SRS, RBS-R, and children’s communication checklist (CCC-2)) or secondary (i.e. CGI or developmental behavior checklist) outcomes. There was no significant difference in the frequency or severity of adverse events between groups.

Overall, although initial small-scale clinical trials show the potential of NAC in reducing irritability in children with ASD (and possibly some aspects of autistic characteristics in the repetitiveness domain), follow-up larger-scale trials so far fail to show impact on ASD core characteristics. Considering the relatively safe side effect profile, NAC may have a positive role in the treatment of irritability in autistic children (85), but so far there is no evidence supporting its use in targeting ASD core symptoms.

### Palmitoylethanolamide

Palmitoylethanolamide is a fatty acid amide with neuroprotective, anti-inflammatory, and anti-nociceptive properties that exert its effect through the peroxisome proliferator-activated receptors (PPAR)-dependent pathway (86). In a case report, Antonucci and colleagues studied efficacy of palmitoylethanolamide on two adolescents with autism. The first was a 13-year-old male with regressive autism and history of eczema and allergy. After treatment with palmitoylethanolamide (Normast®; initially 600 mg daily and then 1200 mg daily) for 1 month, they observed a significant improvement in the CARS-2 score, behavior (i.e. tantrums, stereotyped behaviors, self-talking, outbursts), language abilities, and allergic symptoms (87). The second child was a 15-year-old male with a history of epilepsy. Treatment with Normast for three months led to significant improvement in ATEC score, aggression, cognition, and behavioral skills as well as blood IgE levels. In a randomized controlled trial, Khalaj and colleagues compared risperidone plus palmitoylethanolamide versus risperidone plus placebo over a 10-week period in children (age range of 4–12 years) with ASD (88). Treatment with palmitoylethanolamide resulted in significant improvement in ABC irritability and hyperactivity/noncompliance compared to placebo. The groups did not differ with regard to the side effects.

Taken together, more randomized controlled trials are needed to evaluate the use of palmitoylethanolamide in the treatment of core autism characteristics or associated challenges, as current evidence is still too limited to support its clinical use.

### Pentoxifylline

Pentoxifylline is a xanthine derivative which has an inhibitory effect on inflammatory responses in the body (e.g. by interfering with TNF-alpha effect) (89). In an early open-label trial, Sogame administered pentoxifylline to 36 children (age range of 3–15 years) with ASD or behavioral disorders with organic etiology (90). He observed improvement in maintenance of sameness, language, and relation with others in the majority of the participants. He observed side effects, such as nausea, vomiting, headache, and low blood pressure in a small number of the participants. Nakane reported results of an open-label study of pentoxifylline on 30 children with ASD (91). Children were also on haloperidol. The assessment was based on the parents’ observation. While six children had significant improvement of symptoms, 14 children presented minor improvements. Shimoide administered pentoxifylline to 20 male individuals (age range of 3–22 years) with ASD over a 3-month period and observed improvement in at least two of their three assessments (i.e. patients’ behavior in specific situations, mental developmental scale for young children, Seiken’s critical list for autistic children) (92). Side effects were related to gastrointestinal symptoms. Turek studied the effects of pentoxifylline on 2 children (a 5-year-old boy and a 7-year-old girl) with ASD and 18 with psychosis (93). He observed significant improvement in syllables and words pronunciation of the autistic children. Observed side effects were limited to excitement and sleep disturbances. In the only randomized controlled trial, Akhondzadeh and colleagues compared treatment with risperidone plus pentoxifylline (n = 20) versus risperidone plus placebo (n = 20) (94). Treatment with pentoxifylline led to a significant improvement on ABC in irritability, lethargy/social withdrawal, stereotypic behavior, hyperactivity/noncompliance, and inappropriate speech. Side effects were not significantly different between groups.

Taken together, pentoxifylline may be a beneficial adjuvant therapy for ASD when combined with risperidone, but the evidence is still insufficient, and robust clinical trials are needed.

### Pioglitazone

Pioglitazone is an antidiabetic medication of the thiazolidinedione class that is commonly used for reducing blood glucose in patients with type 2 diabetes mellitus. It exerts its effect mainly through the peroxisome proliferator-activated receptors (PPAR) pathway. Due to the anti-inflammatory properties of this medication, it is suggested to be potentially beneficial in the management of psychiatric disorders (95–97). In an open-label trial, Boris and colleagues administered pioglitazone to 25 children (age range of 3–17 years) with ASD over a 3- to 4-month period and observed a significant improvement in irritability, lethargy, stereotypy behaviors, and hyperactivity as measured by the ABC (98). Improvements in irritability, lethargy, and hyperactivity were significantly associated with age, suggesting younger participants may benefit more from pioglitazone than older children. No significant side effects were observed among the participants.

Most recently, a 10-week randomized controlled trial compared the efficacy of risperidone plus pioglitazone (n = 20) versus risperidone plus placebo (n = 20) (99). This study found a significant improvement in ABC irritability, lethargy/social withdrawal, and hyperactivity/non-compliance after treatment with pioglitazone as compared to placebo. There was no significant difference in the side effects (mostly vomiting and headache) between pioglitazone and placebo groups.

Collectively, initial findings suggest a potential beneficial role for pioglitazone in the management of ASD in children, yet robust clinical trials are needed to provide adequate evidence.

### Riluzole

Riluzole is known as the only beneficial medication for increasing survival in patients with amyotrophic lateral sclerosis (ALS). It mainly exerts its effect by inhibiting presynaptic release of glutamate. It is also shown to have anti-inflammatory effects (100). In a randomized controlled trial, Ghaleiha and colleagues (110) compared risperidone plus riluzole (n = 20) versus risperidone plus placebo (n = 20) over a 10-week period in children (age range of 5–12 years) with ASD. They observed significant improvement in ABC irritability, lethargy/social withdrawal, stereotypic behavior, and hyperactivity/noncompliance. Regarding the side effects, the riluzole group had significantly higher increase in appetite and weight gain. It is noteworthy that a large phase 2 trial studying riluzole effects on 58 participants has been conducted recently (https://clinicaltrials.gov/ct2/show/NCT01661855).

### Spironolactone

Spironolactone as an antagonist for aldosterone receptor has a primary use in the management of condition associated with elevated levels of aldosterone. It is shown to have anti-inflammatory properties, potentially due to its affinity for other steroid receptors (101, 102). In a single case report and hypothesis paper, Bradstreet and colleagues administered spironolactone to a 12-year-old child with ASD and elevated testosterone level, over a 4-week period and observed improvement in irritability (79%), lethargy (83%), stereotypic behavior (60%), hyperactivity (72%), and inappropriate speech (67%) as measured by the ABC (103). They also noted improvement in receptive language.

### Topiramate

Topiramate is an anti-epilepsy medication which is known to have neuroprotective effects (104), exerting through anti-inflammatory and antioxidant effects (105, 106). In the only study in autistic individuals, Rezaei and colleagues compared risperidone plus topiramate (n = 20) with risperidone plus placebo (n = 20) over an 8-week period in children (age range of 3–12 years) with ASD and observed a significant improvement in ABC irritability, stereotypic behavior, and hyperactivity/noncompliance (107). The topiramate group had significantly higher number of somnolence and decreased appetite than the placebo group.

Although initial evidence implies a potentially positive adjuvant role for topiramate, more robust clinical trials are required to determine whether the benefits are consistent and outweigh the side effects.

## Conclusions and Future Direction

While pharmacological interventions are frequently used in the management of concomitant psychiatric and behavior challenges in individuals with ASD, they have limited effect on the core symptoms of ASD. Current evidence on the role of inflammation in the underlying mechanisms leading to autism, or more importantly, to specific subgroups/subtypes of autism (e.g. those with concurrent immunological disorders) (27), has fueled the research on the potential use of agents with anti-inflammatory effects in the management of this condition (108) [as in a recent example of the treatment for a subgroup of individuals with depression (109)]. The literature on anti-inflammatory medications in the management of ASD is still in its infancy, dominated by case reports and open-label studies, with only a small portion of double-blind randomized controlled trials (in which many acted as adjuvant agents combined with risperidone) for which the findings are often not replicated yet. Nevertheless, certain findings imply potential for future investigation with more robustly conducted trials, especially in ASD subgroups such as those with concurrent immunological disorders, developmental regression, or high irritability/behavioral challenges.

This literature implies potential benefits of the following medications for ASD children with severe behavioral challenges (alone or especially when acting as an adjuvant agent with risperidone): amantadine, celecoxib, galantamine, N-acetylcysteine, palmitoylethanolamide, pentoxifylline, pioglitazone, riluzole, and topiramate. A small number of case report and case-series studies suggests a potential role for corticosteroids and ACTH in the management of regressive autism. Flavonoids are suggested by a few open-label studies to be a safe medication to potentially improve behavioral symptoms such as irritability. IVIG might be beneficial in ASD individuals with concurrent immunological disorders. Lenalidomide appeared to be beneficial to ASD individuals with developmental regression and with elevated TNF-alpha. However, it is important to note that the current evidence for efficacy and safety of all these medications in individuals with ASD is still very preliminary and inconclusive. For clinical decision making, safety should be the primary concern. These medications should be considered only when 1) the medication is indicated for the co-occurring medical disorder (e.g. immunological disorders); or 2) standard treatments have been depleted or are insufficient, and the potential benefits are judged to outweigh potential harms [note: N-acetylcysteine has been recommended in the latest clinical care pathway for reducing irritability in autistic individuals (85)]. Current evidence on the role of anti-inflammatory agents in ASD should be interpreted considering the following limitations. First, across all medications reviewed here, there is a lack of rigorous randomized double-blind placebo-controlled trials and very few successful replications so far. The available literature is enriched with case reports and open-label studies. There are a few randomized controlled trials, but they are often of small sample sizes and are being tested in the context of an adjuvant agent (i.e., with risperidone). Therefore, based on the studies that evaluate anti-inflammatory medications as adjuvant therapy, it is unclear whether the effect of an anti-inflammatory medication comes from the medication (or the anti-inflammatory action) itself. Secondly, most clinical trials have focused on short-term effects (about 12 weeks) of the medications. It can be difficult to detect significant changes in a short period of time, and the evidence for long-term efficacy or safety is still lacking. Finally, for most of the medications reviewed here, the exact mechanisms of action (including the biological link between their anti-inflammatory mechanisms and behavior changes of the individual) are not clear, so the interpretation of the findings is challenging, especially when there are other potentially non-immune-mediated mechanisms of action that may contribute to the neurobiological basis of ASD (e.g. the glutamatergic system).

In conclusion, current evidence supporting the efficacy and safety of anti-inflammatory interventions in ASD is still limited. Robust large-scale clinical trials are much needed. Nevertheless, some findings imply that the role for immune-mediated mechanisms in the emergence of autism or autism-related challenges may be specific to a subset of people with autism (27, 111). It may be the case that the greatest potential of anti-inflammatory agents lies in this aspect, in the light of stratified psychiatry and precision medicine.

## Author Contributions

SH, DT, and MC-L designed the study, ran the literature review, prepared the manuscript, and approved the final manuscript.

## Conflict of Interest

The authors declare that the research was conducted in the absence of any commercial or financial relationships that could be construed as a potential conflict of interest.
